# Searching for observational studies: what does citation tracking add to PubMed? A case study in depression and coronary heart disease

**DOI:** 10.1186/1471-2288-6-4

**Published:** 2006-02-16

**Authors:** Hannah Kuper, Amanda Nicholson, Harry Hemingway

**Affiliations:** 1 International Centre for Eye Health, Clinical Research Unit, London School of Hygiene & Tropical Medicine, London, UK; 2 International Centre for Health and Society, Department of Epidemiology and Public Health, University College London Medical School, London, UK

## Abstract

**Background:**

PubMed is the most widely used method for searches of the medical literature, but fails to identify many relevant articles. Electronic citation tracking offers an alternative search method.

**Methods:**

Articles investigating the role of depression in the aetiology and prognosis of coronary heart disease were sought through two methods: a) PubMed, and b) citation tracking where Science Citation Index was searched for all articles which cited ("forward citation tracking") or were cited by ("backward citation tracking") any of the articles in an index review. The number and quality of eligible articles identified by the two methods were compared.

**Results:**

50 articles that were not already included in the index review met our inclusion criteria; 11 were identified through Science Citation Index alone, 8 through PubMed alone, and 31 through both methods. Articles identified by Science Citation Index alone were published in higher impact factor journals, were larger and were less likely to show a positive association.

**Conclusion:**

Science Citation Index identified more eligible articles than PubMed, and these differed qualitatively. Failing to use citation tracking in a systematic review of observational studies may result in bias.

## Background

Highly sensitive methods have been developed for the identification of randomised trials, but there are few guidelines for searching for observational studies. [1] Many research questions can only be addressed through observational studies, for instance the deleterious effects of psychiatric diseases or smoking, and so a review of such a topic would rely on searching for observational studies. PubMed is the most widely used database for searches and is freely available. PubMed may miss many relevant articles and this could influence the conclusions drawn. [2,3] Science Citation Index offers a potential complement to PubMed, particularly in the common situation when at least one review has already been carried out. Once such an index review on the topic in question is identified, Science Citation Index can be used to identify all the articles that cited ("forward citation tracking") or were cited by ("backward citation tracking") the articles in the index review. No previous studies have assessed whether forward citation tracking improves upon a PubMed Search. We sought therefore to compare the number and quality of articles investigating depression and coronary heart disease (CHD) that were identified through Science Citation Index and PubMed.

## Methods

The objective of the literature search was to identify all existing reports of the association between depression and the aetiology and prognosis of CHD that fulfilled the inclusion criteria. Eligible articles were restricted to prospective studies of healthy populations (aetiological) or populations with defined CHD (prognostic) which reported the association between depression and outcome, published before January 2004. Depression was defined by a self-completed scaled questionnaire (such as Centers for Epidemiological Studies on Depression, Beck Depression Inventory), a diagnostic interview, physician diagnosed depression, medication use for depression or self-reported diagnosis of depression. Anxiety alone or composite measures of psychological distress (e.g. vital exhaustion or distress) were not included in this review. Outcomes were defined as fatal CHD and incident non-fatal myocardial infarction, congestive heart failure (aetiology) and mortality from all causes or from coronary disease (prognostic). Patient populations for prognostic studies included post-MI patients, CABG patients as well more general CHD patients, including those with positive angiograms and congestive heart failure. [4] Since coronary disease may also cause depression we chose *a priori *to examine aetiological studies of healthy populations and prognostic studies of coronary disease populations separately. [5]

The index review for citation tracking was a systematic review of prospective studies investigating the role of psychosocial factors in the aetiology and prognosis of CHD. [4] This index review included 56 articles. For the current systematic review the first step was to use Science Citation Index to identify all the subsequent articles that cite any of the 56 articles included in the index review (*forward citation tracking*). Two independent reviewers went through the standard process of screening titles, abstracts and full text versions against the eligibility criteria, with recourse to a third reviewer in the event of a disagreement. As the second step, all the titles of articles in the bibliographies of the 56 articles included in the index review were itemised using the Science Citation Index database (*backward citation tracking*) and the selection procedure was repeated. This search was conducted in May, 2004.

We devised a PubMed search strategy of medical sub heading (MeSH) terms and text words, using the 56 articles included in the index review to develop the search strategy (Table 1). [4] We aimed to produce a search strategy that was both precise and sensitive. This search strategy identified all but six of the articles in the index review, although all 56 articles were indexed in PubMed. Four of these articles did not include "depression" or "depressive" in the keywords, title or abstract. This meant that expanding the PubMed search to identify these four articles would add at least 4,000 unique titles. One of the articles did not mention heart disease in the keywords, title or abstract. Extending the PubMed search strategy to allow identification of the sixth article would have added more than 500 unique titles. The search was conducted using PubMed until the end of 2003 in May, 2004. The procedure for reviewing titles, abstracts and full text articles was repeated as above.

**Table 1:** Summary of terms included in the PubMed search

	**Search terms**	**Number of hits**
1	Mood disorders [MeSH] OR depression [MeSH] AND Heart disease/epidemiology/etiology/mortality/prevention and control/psychology/rehabilitation [MeSH]	1571
2	Mood disorders [MeSH] OR depression [MeSH] AND Cardiovascular diseases/epidemiology/etiology/mortality/prevention and control/psychology/rehabilitation [MeSH] AND myocardial [tw] OR coronary [tw]	985
3.	Personality [MeSH] OR personality tests [MeSH] OR stress, psychological [MeSH] AND Depression [tw] OR depressive [tw] AND Heart disease/epidemiology/etiology/mortality/prevention and control/psychology/rehabilitation [MeSH]	443
4.	Cohort studies [MeSH] OR survival analysis [MeSH] OR proportional hazards model [MeSH] OR case-control studies [MeSH] AND Depression [tw] OR depressive [tw] AND Heart disease/epidemiology/etiology/mortality/prevention and control/psychology/rehabilitation [MeSH]	1005
5.	Antidepressive agents/adverse effects [MeSH] AND Heart disease/epidemiology/etiology/mortality/prevention and control/psychology/rehabilitation [MeSH] AND Cohort studies [MeSH] OR survival analysis [MeSH] OR proportional hazards model [MeSH] OR case-control [MeSH]	43
**Total number of hits**		**2501**

The publication year, sample size and journal impact factor were recorded for the eligible articles. The reported effect estimate for the association between depression and CHD of each study was classified as: positive (i.e. statistically significant positive association or a relative risk ≥2), null (i.e. statistically non significant association or a relative risk >0.5–<2) or negative (i.e. statistically significant inverse association or a relative risk ≤0.5) by two reviewers, with arbitration by a third reviewer in the event of disagreement. The Kruskal-Wallis and Chi-square tests were carried out to test for differences in the characteristics of articles (i.e. year of publication, journal impact factor, type of study and the classification of outcome) identified by Science Citation Index Alone, PubMed or through both strategies.

## Results

Science Citation Index identified more unique titles than PubMed (2906 and 2501 respectively). Science Citation Index also identified more abstracts for review (832 and 794) and articles for review (345 and 254). Eleven articles were identified through Science Citation Index alone (7 forward, 4 backward), 8 through PubMed alone, and 31 through both methods (Table 2). Citation tracking added approximately 2 person-weeks of reviewer's time to the review.

**Table 2:** Eligible articles identified through the Science Citation Index and PubMed search strategies

	Articles identified through Science Citation Index	
Articles identified through PubMed	Yes	No
Yes	31	8
No	11	-

Nine of the 11 articles that were identified through Science Citation Index alone were within the PubMed database. They were not detected by the PubMed search because they did not include depression or depressive in key words or MeSH headings (n = 6) and/or did not include the relevant heart disease terms (n = 5).

Articles identified by Science Citation Index alone were larger, more likely to be published in higher impact factor journals and were less likely to show a positive association (Table 3). Articles identified by both PubMed and Science Citation Index were published more recently, although the difference was not statistically significant.

**Table 3:** Comparison of articles identified through Science Citation Index and PubMed searches until the end of 2003

**Search method(s) that identified article**	**PubMed and Science Citation Index (n = 31)**	**Science Citation Index only (n = 11)**	**PubMed only (n = 8)**
Mean (s.d.)	1999 (6.4)	1996 (7.9)	1997 (9.9)
Median (range)	2002 (1969–2003)	2001 (1982–2003)	2001 (1973–2002)
**Impact factor**			
Mean (s.d.)*	4.2 (2.8)	9.5 (9.8)	3.9 (2.6)
Median (range)	3.2 (1.1–10.5)	3.8 (2.0–31.7)	2.8 (1.3–7.6)
**Study Type**	8 aetiologic 23 prognostic	3 aetiologic 8 prognostic	2 aetiologic 6 prognostic
Classification of outcome**	25 positive 6 null	5 positive 6 null	6 positive 2 null
**Sample size: aetiologic studies**			
Mean (s.d.)	4502 (3331)	5758 (4397)	1101 (1089)
Median (range)	3269 (76–9758)	7217 (817–9239)	1101 (332–1870)
**Sample size: prognostic studies**			
Mean (s.d.)	525 (626)	832 (925)	399 (441)
Median (range)	344 (30–2885)	552 (58–2320)	288 (66–1250)

* Four articles were published in journals with no impact factor (1 both methods, 1 Science Citation Index only, 2 PubMed only).

**Statistically significant result at the p < 0.05 level.

## Discussion

In this case study, Science Citation Index identified more eligible articles than PubMed and these articles were published in higher impact journals. Articles identified through the Science Citation Index were less likely to show positive results. This may be because articles that reported no association between depression and CHD may emphasise other relationships explored in the article and so would not include the appropriate text words or MeSH headings for depression in PubMed. Indeed, nine of the eleven articles identified only through the Science Citation Index were in the PubMed database but did not include relevant indexes. The inadequate indexing using MeSH intervention terms and the incomplete reporting of collected data for observational studies are consistent with the findings of an earlier report. [6]

It is not surprising that citation tracking improved upon PubMed. Forward citation tracking allows the accumulation of multiple searches carried out by different publishing research groups using different (unreported) search methods. Citation tracking is wholly independent of the need to specify search strategies or use MeSH headings, which are a potential limitation of MEDLINE. However, starting the citation tracking with an index review that included a smaller number of articles would mean that the search would take less time but may yield fewer eligible articles. The relative efficiency and time taken by the two methods may therefore depend on the index review used in citation tracking.

It is well known that existing search methods fail to identify the complete set of eligible articles [2]; Two previous systematic reviews of depression and CHD identified few eligible articles. [7,8] Conclusions drawn from a systematic review may be influenced by the number of eligible articles identified and the search strategy used. [3] If, as we suggest, the characteristics of articles identified through PubMed and citation tracking differ then failing to use citation tracking in a systematic review of observational studies may result in bias. The present case study does not prove that citation tracking improves upon PubMed in other observational settings and the results cannot be generalised to searches for clinical trials, but we suspect that the chances of funding such bibliographic research are low. The gains from citation tracking or another search method depend, of course, on the sufficiency of the rest of the search strategy (both electronic and non-electronic) used in the systematic review and reviewers should focus on searching exhaustively for relevant articles, as well as on using appropriate search methods.

## Conclusion

Although Science Citation Index is only available by subscription, since citation tracking involves only a modest additional work load (in this case approximately two person weeks) and may offer an opportunity to reduce bias, we propose that the onus should be on systematic review protocols to justify situations where citation tracking has *not *been used.

## Abbreviations

CHD – Coronary Heart Disease

MeSH – Medical Sub Heading

s.d. – Standard Deviation

## Competing interests

The author(s) declare that they have no competing interests.

## Authors' contributions

HK and AN were responsible for reading the titles, abstracts and articles and determining their eligibility, with recourse to HH in the event of a disagreement.

HK was responsible for drafting the article, and AN and HH were responsible for revising it critically for important intellectual content. All authors read and approved the final manuscript.

## Pre-publication history

The pre-publication history for this paper can be accessed here:


